# Emerging Plant Intoxications in Domestic Animals: A European Perspective

**DOI:** 10.3390/toxins15070442

**Published:** 2023-07-04

**Authors:** Andras-Laszlo Nagy, Sabrina Ardelean, Ronan J. J. Chapuis, Juliette Bouillon, Dalma Pivariu, Alexandra Iulia Dreanca, Francesca Caloni

**Affiliations:** 1 Department of Biomedical Sciences, Ross University School of Veterinary Medicine, Basseterre P.O. Box 334, Saint Kitts and Nevis; nagyandras26@gmail.com (A.-L.N.); rchapuis@rossvet.edu.kn (R.J.J.C.); 2 Department of Clinical Sciences, Ross University School of Veterinary Medicine, Basseterre P.O. Box 334, Saint Kitts and Nevis; sabrinaardelean@gmail.com (S.A.); jbouillon@rossvet.edu.kn (J.B.); 3 Department of Toxicology, Faculty of Veterinary Medicine, University of Agricultural Sciences and Veterinary Medicine Cluj-Napoca, Calea Manastur 3-5, 400372 Cluj-Napoca, Romania; dalma.pivariu@usamvcluj.ro (D.P.); alexandra.dreanca@usamvcluj.ro (A.I.D.); 4 Department of Environmental Science and Policy (ESP), Università degli Studi di Milano, Via Celoria 10, 20133 Milan, Italy

**Keywords:** animals, emerging plants, phytotoxin, poisoning, toxicity

## Abstract

Exposure to phytotoxins that are present in imported ornamental or native plants is an important cause of animal disease. Factors such as animal behaviors (especially indoor pets), climate change, and an increase in the global market for household and ornamental plants led to the appearance of new, previously unreported plant poisonings in Europe. This has resulted in an increase in the incidence of rarely reported intoxications. This review presents some of the emerging and well-established plant species that are responsible for poisoning episodes in companion animals and livestock in Europe. The main plant species are described, and the mechanism of action of the primary active agents and their clinical effects are presented. Data reflecting the real incidence of emerging poisoning cases from plant toxins are scarce to nonexistent in most European countries due to a lack of a centralized reporting/poison control system. The diversity of plant species and phytotoxins, as well as the emerging nature of certain plant poisonings, warrant a continuous update of knowledge by veterinarians and animal owners. The taxonomy and active agents present in these plants should be communicated to ensure awareness of the risks these toxins pose for domestic animals.

## 1. Introduction

Poisonings by phytotoxins, such as alkaloids, glycosides, oxalates, proteins, and others [1,2], exert species-specific toxicity and are frequently reported in animals [3]. Temperature and geographical distribution are factors that can affect toxicity within plant species [4]. The impact of climate change may play an important role in the emerging issues related to exposure to some plant toxins.

The toxicological effects depend not only on the characteristics of the plant, but may also be influenced by its growth stage, the amount ingested, and, at times, the part of the plant ingested [3].

Both livestock and companion animals are affected by plant poisoning [2,5,6,7,8].

Most of the poisoning cases involving dogs and cats are accidental [9] and are mainly related to household and ornamental plant exposures [7]. Food containing plant material also presents a potential source of intoxication [6].

Although dogs are most frequently involved in poisoning cases, cats appear to be more susceptible to plant toxins [7], and young animals are more frequently affected than adult ones [5].

Large animal plant poisoning rarely occurs; it usually happens if no other feed is available or when hay or silage are contaminated [4,10]. Among livestock, cattle and sheep are the most affected species [11].

A recent analysis of European literature [8] reported that the proportion of inquiries to the Poison Control Centre for pet poisonings by plants was between 5–6% and 11% [2,12,13,14]. Ten years of epidemiological data spanning from 2000 to 2010 from the Poison Control Center of Milan showed that poisonings involving plants represented between 1.7% (2004) and 8.9% (2009) [15] of the inquiries, while data collected between January 2015 and March 2019 showed that 10.19% of calls were for poisonings involving plants [2]. Data collected at the Centre National d’Informations Toxicologiques Vétérinaires (CNITV) in Lyon showed that plants were involved in 11% of the cases at this institution [16].

Recent studies revealed a new epidemiological trend of plant toxicoses mainly related to the current global trade in ornamental plants [2]. These new, previously unreported or rarely reported poisoning cases occurred especially in companion animals.

The present review aimed to update current knowledge regarding plant poisonings, emerging and not, in Europe, with a focus on describing the main plant species involved (Table 1), their active compounds and their mechanism of action, and the clinical effects associated with toxicosis.

## 2. Plants Involved and Their Description

### 2.1. Allium cepa and Allium sativum

*Allium cepa* (onion) and *Allium sativum* (garlic) belong to the *Amaryllidaceae* family. They are pungent aromatic herbs that sprout from bulbs, and their leaves are basal and long with sheathing bases. Their flowers are in umbels and the fruits are three-lobed loculicidal capsules [17] (pp. 753–757).

Onion is a biennial or perennial plant, frequently harvested in its first growing season, while garlic is a perennial plant that is usually grown as an annual crop. Other important potentially toxic *Allium* species include *Allium porrum* (leek) and *Allium schoenoprasum* (chive). *Allium* case reports in dogs and cats are frequently reviewed [33]. Fresh, spoiled, powdered, and cooked plants are poisonous, with cumulative toxic effects [21,33] (pp. 753–757).

The active compounds of these plants are disulfides and thiosulfates, especially n-propyl disulfide [21] (pp. 753–757). *Allium* species are rich in alk(en)ylcysteine sulfoxides. Traumatic destruction of the plant tissues will allow for the enzymatic conversion of sulfoxides to less stable compounds, which are further converted into an array of sulfides, disulfides, trisulfides, and thiosulfonates by nonenzymatic reactions [9,18,21] (pp. 753–757). Further biotransformation reactions may take place in the liver. Sulfides from the *Allium* species can form highly reactive oxidants. Unsaturated 1-propylene is the most important hemolytic agent. The hemolytic effect occurs when the level of oxidants in red blood cells is high enough to overwhelm the capacity of their antioxidant metabolic pathways [17,21,34] (pp. 753–757). Dogs’ erythrocytes are predisposed to oxidative damage due to their low catalase level. Cats are even more predisposed to oxidative damage because their hemoglobin contains eight sulfhydryl (thiol) groups compared with a dog’s hemoglobin, which contains only four. A cat’s hepatic capacity is also much lower for glucuronidation [35].

The oxidative effect of the active ingredients induces methemoglobin and Heinz body formation with consecutive hemolytic anemia. Oxidants may also affect the erythrocyte cytoskeleton, resulting in eccentrocyte formation [17,36]. Heinz body and eccentrocyte formation causes membrane rigidity and red blood cell lysis [37,38,39].

The toxic dose of onions is 15 to 30 g/kg in dogs and 5 g/kg in cats. Toxicity can occur via the consumption of a single large dose or the repeated ingestion of small amounts [17]. Inborn errors of metabolism, such as deficiencies in Glucose-6-phosphate dehydrogenase or zinc, increase susceptibility to *Allium* species toxicity. Dogs with hereditary high-erythrocyte-reduced glutathione and potassium concentrations are also more susceptible to the hemolytic effects [40].

The clinical signs of *Allium* toxicity are hemoglobinuria, depression, and anorexia. Weakness, ataxia, tremors, and pale mucous membranes can also manifest. Increased heart and respiratory rates are frequently observed. Vomiting, diarrhea, icterus, and abdominal pain are other common symptoms reported. Hemoglobin and possibly hemosiderin urinary casts may be seen. Usually, the animal’s breath or body has a very strong onion odor [17,21] (pp. 753–757).

Clinical pathology findings indicate intravascular and extravascular hemolysis. The most important findings are Heinz body anemia, eccentrocytosis, hemoglobinemia, hemoglobinuria, hyperbilirubinemia, and methemoglobinemia [17,34].

The most common pathologic features encountered are a pungent body odor, splenomegaly, pale liver, and dark kidneys. The plants are sometimes present in the stomach. Microscopically, mild-to-moderate hepatic necrosis, renal tubular nephrosis (pigment nephrosis) with tubular casts, and splenic hematopoietic foci may also be observed [21] (pp. 753–757).

The diagnosis of *Allium* poisoning is based on a combination of history, clinical signs, and the confirmation of a Heinz body hemolytic anemia [17].

In recent years, several case reports regarding onion/garlic poisoning in dogs in several European countries were published. In Turkey, Altinok-Yipel et al. (2016) reported the case of a 2-year-old, 2.5 kg male Yorkshire terrier, who displayed anorexia, weakness, depressed mentation, and vomiting after ingesting a meal with meat and onion. Hemolytic anemia and dark-colored urine (hemoglobinuria) were detected at the hematological examination and urinalysis. The dog fully recovered following treatment [41].

In Spain, Guitart et al. (2008) reported Heinz body anemia in two dogs after the ingestion of Catalan spring onions. In both cases, severe Heinz body anemia and the presence of eccentrocytes in new methylene-blue-stained blood smears were noted. Both animals fully recovered following rigorous therapy [42].

Jayson et al. (2018) reported a case of Heinz body hemolytic anemia associated with leek (*Allium ampeloprasum*) consumption in a South American coati at the London Zoo in the United Kingdom. Leeks were introduced into the animal diet for 2–5 days prior to the initial presentation. Supportive care, along with a blood transfusion, resulted in the immediate improvement of the clinical signs [43].

### 2.2. Anthurium spp.

*Anthurium* spp., or flamingo plant, is native to South America and belongs to the *Araceae* family. There are more than 950 species distributed throughout the neotropics [44]. It is a monocotyledonous perennial plant with a preference for warm, shady, and humid climates [45]. Its most characteristic feature as an ornamental is its brightly colored spathe leaf, and the protruding inflorescence called the spadix [45]. It has large, fleshy, shiny, dark green leaves and bright red or pink spathe flowers with yellow spadices [2,45]. All parts of the plant are toxic. It is considered a plant with mild-to-moderate toxicity [46].

The active compounds are insoluble calcium oxalates that cause oral irritation and edema of the mucosa, as well as drooling, gagging, and vomiting if any part of the plant is chewed [8]. Other common symptoms include difficulty swallowing, pawing at the face, and head shaking. Cases were reported in dogs and cats [2,8,15].

### 2.3. Arum italicum

*Arum italicum*, known as Italian lords-and-ladies or Italian arum, belongs to the *Araceae* family. It is native to Europe, North Africa, and western Asia, and is used ornamentally [19]. The plant grows in shaded areas, with green foliage over winter, inflorescence in spring, and the absence of foliage in the summer when only red and orange berries remain [19].

*A. italicum* may contain four to five types of phytotoxins: the potentially hemolytic saponin arin, a coniine-like alkaloid and nerve toxin, the calcium oxalate forming topically irritating raphides, and a cyanogenic glycoside [19].

*A. italicum* is reported to be unpalatable for mammals, including guinea pigs, rats, mice, dogs, badgers, and pigs [19]. There is scarce information about the toxicity of this plant. *Arum* spp. containing oxalate and proteolytic enzymes were reported in 2021 as a source of intoxication in two dogs in Italy [2].

### 2.4. Cycas revoluta

*Cycas revoluta*, known as sago palm or king sago, belongs to the *Cycadaceae* family, is native to tropical and subtropical regions in Japan, and is used as an ornamental plant [8,20]. While long used as a food source for humans, it must be processed for detoxification before consumption [20]. Indeed, cycad species contain three types of phytotoxins, namely, the azoxyglycosides, β-methylamino-l-alanine (BMAA), and an unidentified high-molecular-weight compound [8,20]. The azoxyglycosides include cycasin, neocycasin, and macrozamin. Cycasin and neocycasin are cyanogenic glycosides, though they are considered pseudocyanogenic as they have low cyanogenic potential reported in humans [47]. Azoxyglycosides are amino sugars, with a variable sugar component that binds each azoglycoside to methylazoxymethanol (MAM) with a glycosidic bond. The hydrolysis of azoglycosides is required to release the toxic compound MAM. This occurs in the intestines of mammalian species, and due to enterohepatic circulation, the toxic potential of MAM is enhanced. The MAM alkylates DNA and RNA [48], and is neurotoxic, carcinogenic, mutagenic, teratogenic, and hepatotoxic [49]. The β-N-methylamino-L-alanine is an amino acid associated with neurodegeneration in animal models and possibly in cattle [20]. The unidentified high-molecular-weight compound was associated with neuropathy in cattle [20].

The toxins, though present in all parts of the plant, are more concentrated in the seeds [8,20].

Cycad toxicosis involves mainly dogs [2,8,13,15], but cases of toxicosis in cats were reported and warrant further investigation [48,50]. In dogs, though the consumption of any part of the plant can lead to clinical signs, the ingestion of the seeds is more commonly reported and leads to more severe toxicosis [2,20,51]. A mortality rate as high as 67% was reported in dogs, with increased odds with the consumption of seeds [51]. Clinical signs can be detected 4 to 24 h post-exposure and include an abnormal rectal temperature, inappetence, lethargy, hypersalivation, emesis, hematemesis, regurgitation, abdominal pain, diarrhea, constipation, melena, hematochezia, tremor, weakness, proprioceptive deficits, seizure, coma, icterus, petechia, and ecchymoses [20,48,51]. Abnormalities in blood biochemistry that are consistent with liver damage (i.e., increased liver enzymes activity) and liver failure (i.e., hyperbilirubinemia, abnormal glycemia, hypocholesterolemia) can be detected within 72 h post-exposure [20,51]. Other abnormalities reported in blood work include hypoproteinemia/albuminemia, azotemia, anemia, increased coagulation time, and thrombocytopenia [20,51]. Urinalysis can show glycosuria, bilirubinemia, and hematuria [48]. Among these findings, elevated alanine transaminase activities and thrombocytopenia were associated with higher mortality [51].

### 2.5. Lantana camara

*Lantana camara* (red sage, yellow sage) is a weed of notoriety and an ornamental plant from the *Verbenaceae* family [22,52]. It is an invasive weed native to the tropics and subtropics [21,53,54] (pp. 1201–1208). It is a broadleaf evergreen shrub with erect stems and small tubular-shaped flowers of white, red, yellow, orange, or intermediate color [21,55] (pp. 1201–1208). The leaves have a strong odor when crushed. The fruits are black, globose, fleshy drupes with two stones [21] (pp. 1201–1208).

There is a great variation in the toxicity among cultivars, where red- and yellow-flowered types are of high risk for animals, pets, and livestock [21] (pp. 1201–1208).

*Lantana camara* is toxic to dogs, cats, horses, ruminants, and many other species [2,53,56].

The active compounds of *Lantana camara* are pentacyclic triterpenoids. The foliage and ripe berries contain the highest quantity of toxins [57]. The first recognized toxic triterpenoid was lantadene A [22,56]. Pentacyclic triterpenoids are hepatotoxic and are irritants of the gastrointestinal tract.

The primary problem caused by *Lantana* is obstructive cholangitis [21] (pp. 1201–1208). *Lantana camara* causes steatosis, which leads to hepatocyte swelling and cholestasis [58]. In addition, the triterpenoids damage bile canalicular membranes and microvilli, leading to decreased ATPase activity and the blockage of bile flow [21] (pp. 1201–1208). Obstructive cholangitis induces bilirubin and phylloerythrin retention, leading to photosensitization in ruminants [21,56] (pp. 1201–1208).

In dogs and cats, the initial clinical signs of *Lantana camara* poisoning are anorexia, weakness, depressed mentation, and constipation, which occur within several hours following plant ingestion. In 48–72 h after ingestion, melena; icterus; and marked elevations of serum bilirubin, ALT, GGT, and alkaline phosphatase levels are observed. Dogs usually do not ingest a fatal dose [57,59]. If not treated immediately, the clinical condition of the patient can deteriorate, resulting in death within 1–3 weeks after exposure. Ingestion of a high, fatal dose causes death in 2–4 days [21,53,57] (pp. 1201–1208).

In ruminants and horses, the chronic form of the disease develops over several weeks and manifests mainly as photosensitization [21] (pp. 1201–1208).

The pathological change routinely observed in affected animals is the congestion of the gastrointestinal mucosa due to the irritating effect of *Lantana*. Hepatic lesions include hepatomegaly and orange discoloration of the liver. Microscopically, centrilobular necrosis, ductular reaction, and even portal fibrosis (in chronic cases) are reported. Usually, the gallbladder is enlarged and edema of the wall is evident microscopically [21] (pp. 1201–1208).

### 2.6. Lilium spp.

*Lilium* is a large genus, with over 100 recognized species and many hybrids, and is native to the temperate regions of the northern hemisphere [21] (pp. 771–773). It is an ornamental flower, frequently cultivated as a house plant (potted plant) or garden plant for its unique esthetic, medicinal, and edible values [23,60].

Lillies are bulbous perennial plants, with erect stems, alternate or whorled leaves, and funnelform, cup-shaped, or bowl-shaped flowers of white, yellow, orange, red, or maroon colors [21] (pp. 771–773).

Cats are the most sensitive species to poisoning by plants from the genus *Lilium*. These plants are nephrotoxic to cats, causing acute renal failure in this species [8,23,61]. Although less typical, pancreatic degeneration and pancreatitis can also occur in a subset of cats [23]; therefore, veterinarians should consider pancreatitis as a possible outcome of exposure to Lilium [23]. Cats frequently ingest the leaves and flowers of these plants [23]. Consumption of a small amount, such as one or two leaves or one whole flower, can cause toxicosis in cats [23]. Dogs do not develop nephrotoxic signs after exposure, but mild gastrointestinal signs were described with the exposure to large doses [2,62].

The toxic compounds of lilies are different steroidal glycoalkaloids (SGA) and steroidal saponins. The active compounds are found in the aqueous extract of the plant material [23,61]. The steroidal glycoalkaloids are the principal cytotoxic constituents of the plant extract [61], while other toxins are believed to cause pancreatic damage [23].

Different species of *Hemerocallis* (daylilies) are also nephrotoxic to cats and will induce similar clinical signs as *Lilium* spp. [21] (pp. 771–773).

The clinical signs associated with exposure in cats are anorexia, vomiting, polydipsia, and depression [23], followed by typical signs of acute renal failure 12–72 h after ingestion. Typically, an initial phase of polyuric renal failure with dehydration is followed by a phase of anuric renal failure [63]. Seizures, as a consequence of severe uremia, were also reported [23]. Cats with anuric renal failure usually die within 3 to 7 days after ingestion [63].

Clinical pathology values are characteristic of acute renal failure, including increased blood urea nitrogen, creatinine, phosphorus, and kalemia, as well as proteinuria, glucosuria, and isosthenuria or elevated creatinine kinase activity [21,23,63] (pp. 771–773).

The most important microscopic lesions observed in cats exposed to lilies were degeneration and necrosis in the proximal convoluted tubules of the kidneys [21,23] (pp. 771–773). In Europe, several case reports involving *Lilium* species were published, with cases of poisoning in cats reported in Hungary, Switzerland, the United Kingdom, Italy, and France [8]. According to the data from the Milan Poison Control Centre, *Lilium* spp. are some of the most frequently involved plants in cases of animal poisoning in Italy [64].

Recently, Ozaki et al. described a fatal case of oriental lily poisoning in a meerkat. The meerkat presented with clinical signs that were similar to those described in cats and died 40 h post-ingestion [65].

### 2.7. Melia azedarach

*Melia azedarach* or Chinaberry tree, also known as white cedar, pride of India, or Indian lilac, is a deciduous tree native to Asia, belongs to the *Mahogany* or *Meliaceae* family, and is used as an ornamental plant worldwide [9,24,25].

The leaves of the plant are pinnately compound, alternate, and with fragrant flowers ranging from white to lavender. The flowers are arranged in large axillary panicles originating from the leaf axils. The fruits are small and fleshy seedpods or drupes, round-to-ovoid-shaped, and are initially green and turn pale yellow when ripe [9,21,24] (pp. 825–829). The bark is thin, finely furrowed, and grayish-brownish colored [24].

Cases of poisoning with *Melia azedarach* were reported in horses, cattle, sheep, goats, pigs, dogs, rabbits, rats, guinea pigs, and poultry [24]. Most of the poisoning cases occur in autumn and winter when the fruits ripen [25].

The active compounds in the chinaberry are represented by several tetranortriterpenes called meliatoxins A1, A2, B1, and B2, which are present in high concentrations in the fruits. In the bark of the tree, other toxins, including havanensin-class limonoids, such as toosendanin, are present [21,24] (pp. 825–829).

The toxic dose is not clearly established, as the toxicity of the plant could be different according to the geographic location, climatic conditions, or stage of growth [24,25]. Previous case reports showed that a small dog can die after the ingestion of 5 to 6 drupes [24].

Meliatoxins act as enterotoxins and neurotoxins, but their mechanism of action is not fully understood [9,24]. The symptoms usually appear within 1 to 2 h after ingestion and are characterized by anorexia, vomiting, or diarrhea (often with blood), as well as colic. These gastrointestinal signs are followed by neurological signs, such as ataxia, excitement, seizures, depression, paresis, coma, and death due to respiratory failure [9,24,25].

The lesions induced by chinaberry poisoning are unspecific; gastrointestinal irritation and degenerative changes of the liver and kidney are reported [24,25].

Case reports of chinaberry poisoning in dogs are rare, with only a few cases being published in the literature in recent years [15,25,66].

### 2.8. Nandina domestica

*Nandina domestica*, also known as nandina, sacred bamboo, or heavenly bamboo, is an ornamental perennial deciduous shrub from the plant family *Berberidaceae* [26,67]. The plant generates terminal conical clusters of white or pink flowers in early summer and bright red berries from fall to spring [8,26].

This plant contains cyanogenic glycosides that can cause cyanide poisoning. A recent study revealed that the leaves are strongly cyanogenic throughout the year, and the green fruits are strongly and rapidly cyanogenic, while most ripe fruits are weakly and slowly cyanogenic [67]. When the plant is ingested and masticated, cellular compartmentation, which normally keeps cyanogenic glycosides separated from β-glycosidases, is disintegrated, allowing the cyanogenic glycosides to be hydrolyzed by the β-glycosidases and then cleaved by lyases. This results in the production of hydrogen cyanide, also known as prussic acid [26,27,68,69].

Cyanide inhibits mitochondrial respiration by initially binding to copper in the binuclear center of the cytochrome oxidase a3 [8,27,68]. Therefore, aerobic cellular metabolism is interrupted, and cellular hypoxia, as well as an energy deficit (i.e., lack of ATP formation), ensues [68,70]. Additional mechanisms include the inhibition of multiple enzymes and sulfhydryl compounds, an increase in cellular calcium level, modulation of NMDA receptors, and stimulation of neurotransmitter release [70].

The lethal dose of hydrogen cyanide ranges from 2 to 2.5 mg/kg body weight for most species [27], and acute toxicity can cause death within a few hours. The plant also contains protoberberine and berberine alkaloids [2], whose toxic significance is unknown. The alkaloid berberine carries anticholinesterase activity [8] and was shown to cause emesis in cats, as well as salivation, nausea, diarrhea, emesis, muscular tremor, and sometimes paralysis in dogs [71]. Repeated oral administration (100 mg/kg for 8–10 days) caused the death of all cats [71]. Clinical signs include the typical dark/cherry-red mucous membranes associated with the elevated oxygen saturation of venous blood, as well as signs associated with cellular hypoxygenation: vomiting, abdominal pain, ataxia, weakness, increased temperature, tachycardia, hypertension, respiratory failure, seizures, and shock [26,72]. Although there is no report of intoxication in ruminants, they are considered at risk of intoxication and may even be more susceptible than other species, as rumen microorganisms possess the enzymes β-glycosidases and hydroxynitrile lyase [27,68]. Additionally, the neutral pH of the rumen favors the enzymatic reactions that produce hydrogen cyanide [68]. In Italy, a case report of intoxication in dogs was recently reported [2,8]. *Nandina domestica* intoxication has also been reported in young children (<5 years old) and clinical signs were mostly limited to gastrointestinal signs (e.g., nausea, vomiting, diarrhea) [26].

### 2.9. Persea americana

Avocado (*Persea americana*) is an evergreen tree in the laurel family (*Lauraceae* family). The tree is approximately 30 m tall with slightly furrowed brown bark [21] (pp. 744–747). The leaves are alternate rounded, ovate, and oval in shape; they range in length from 8 to 25 cm with one primary vein; and the flowers are greenish-yellow arranged in panicles [21,73] (pp. 744–747). The fruit is a berry with one large stone, which takes up around 10 to 25% of the weight of the avocado fruit [73]. A variety of avocado cultivars exist, with the fruits being the nutritional part [74].

The active compound responsible for the toxicity is persin [18,21] (pp. 744–747). Persin is an acetogenin that is derived from the biosynthesis of long-chain fatty acids and possesses a structure similar to linoleic acid [75].

Persin is a naturally occurring anti-fungal toxin found in both the fruit and leaves of *Persea americana* [18,76]. This compound is also known to have insecticidal properties. Unfortunately, persin can also be variably toxic to animals. Dogs and cats are rarely affected by persin [77].

Clinical signs in dogs are characterized by mild gastrointestinal symptoms including anorexia; gastrointestinal tract irritation; vomiting; diarrhea; abdominal swelling; and foreign body obstruction in the esophagus, stomach, or intestines if a large avocado seed is ingested [18,77].

Dogs that ingest large amounts of avocado can be at risk of pancreatitis, respiratory distress, hydropericardium, and even death [18,74].

Although the toxicity for dogs is thought to be low, myocardial damage on histopathology of the cardiac tissue, as well as congestion and inflammation in the liver and kidneys, were reported in two dogs. These post-mortem findings were deemed highly similar to those in goats, sheep, and horses poisoned by avocados [78].

Recently, a case of poisoning in a cat was reported in Italy [2]. In cats, the clinical signs are similar to those described in dogs [18,77].

### 2.10. Prunus laurocerasus

Cherry laurel is a plant from the *Prunus* genus of the *Rosacea* family, cultivated as a decorative plant, and occasionally as a hedge plant. It is an evergreen shrub or a small tree with reddish-black berries and clusters of tiny, fragrant white flowers, and is native to the temperate climates of the Northern Hemisphere [21] (pp. 1084–1086).

The major active ingredients of *Prunus laurocerasus* are cyanogenic glycosides, such as prunasin, sambunigrin, and amygdalin [79].

These compounds are present in several *Prunus* species, including the cherry laurel (*Prunus laurocerasus*), peaches, cherries, apricots, plums, and nectarines [80].

In the gastrointestinal tract, following the hydrolysis of cyanogenic glycosides, hydrogen cyanide is formed [81]. Hydrogen cyanide (HCN) primarily inhibits cytochrome C oxidase (CcOX), inhibiting mitochondrial respiration and leading to cellular hypoxia by preventing hemoglobin in red blood cells from releasing oxygen to the tissues [2,82,83,84]. Ruminants, specifically goats, are more prone to develop this intoxication due to the ruminal hydrolyzation potential. Interestingly, intoxications from this plant were reported in dogs and cats, despite the acidity of monogastric stomach contents limiting the hydrolysis and release of HCN [85].

Most dogs and cats that consume plants containing cyanogenic glycosides exhibit gastrointestinal symptoms, including vomiting, diarrhea, and abdominal pain. Depending on the extent of exposure, there is also a chance of constipation and gastrointestinal obstruction. Particular signs of cherry laurel poisoning include trembling, shaking, high excitability, convulsions, difficulty breathing, cardiac arrhythmias, frothing at the mouth, and profuse drooling [80].

There are numerous case reports detailing goat intoxication. All cases reported acute clinical signs, including weakness, depressed mentation, hypersalivation, seizure-like activity, mouth breathing, dyspnea, and lateral recumbency. The diagnoses were made postmortem following the finding of large quantities of cherry laurel leaves in the rumen [28,29]. Less than 450 g of wilting cherry tree leaves can be fatal in goats [28,29].

A fatal case of *Prunus lauracerasus* poisoning in a goat was recently reported by the Milan Poison Control Centre [2].

### 2.11. Sorghum spp.

*Sorghum* spp. (*Poaceae* family) are among the most widely used pastures for animals and cereals for humans. *Sorghum* spp. play a significant economic role in the production of grain, hay, silage, and forage. It is the fifth most important cereal grown for forage, grain, and biofuel [86]. Nevertheless, sorghums, sorghum–sudangrass crosses, and sudangrasses may be poisonous if grazed or improperly fed to cattle and small ruminants [21,85] (pp. 888–890).

The major active compounds of *Sorghum* plants are represented by cyanogenic glycosides, but they are also nitrate accumulators [21] (pp. 888–890). Many factors, such as plant age, irrigation, and the plant part influence the cyanide and nitrate levels in these plants [86]. For example, compared with stems, leaves have more toxins [87]. Soil has an important influence on toxin accumulation; soils that are rich in nitrogen and poor in phosphorus have a higher cyanogenic potential [88].

All animals are susceptible to this type of intoxication, but the ability of ruminal microflora to rapidly hydrolyze cyanogenic glycosides makes ruminants particularly at risk of cyanide intoxication [85]. All sorghums produce cyanogenic glycosides during their growing stage. The most important cyanogenic glycoside in *Sorghum* plants is dhurrin, which is a chemical that is non-toxic when unaltered, but following metabolism, is converted into hydrogen cyanide via a two-step enzymatic hydrolysis [89]. The HCN level in plants influences their toxicity: cyanide levels between 0 and 25 mg/100 g of dry weight have been deemed safe for grazing, levels between 50 and 75 mg/100 g are considered mildly toxic, and above 100 mg/100 g as extremely toxic [30].

After the ingestion of the plant, the HCN is released in the rumen, absorbed into the bloodstream, and transported to the tissues. In the cytochrome oxidase system, cyanide ions and ferric irons interact to block the electron transport chain. HCN does not inhibit hemoglobin from carrying oxygen, but blocks the utilization of oxygen from hemoglobin, resulting in cellular anoxia and rapid death [85,89,90].

An animal poisoned with cyanide exhibits acute clinical signs of tachypnea, tachycardia, gasping, muscle twitching or anxiety, trembling, foaming from the mouth, cyanosis, and spasms or convulsions; death results from respiratory paralysis [30,91]. There are no characteristic postmortem lesions, but bright red venous blood and subendocardial and subepicardial petechial hemorrhages are typically present.

In August 2022 in northwestern Italy, more than 50 cows died after consuming young *Sorghum* plants high in cyanogenic glycosides. The excessive accumulation of cyanogenic glycosides was attributed to excessive drought during the summer months [92].

### 2.12. Spathiphyllum spp.

*Spathiphyllum* spp., or peace lily, are tropical plants and members of the *Aracae* family. They are evergreen perennial plants with large, dark green, glossy leaves [21] (pp. 131–144). The flowers are produced in a spadix, surrounded by a 10–30 cm long, white, yellowish, or greenish spathe. They are common ornamental plants.

The active compounds of this plant are insoluble calcium oxalate crystals that are aggregated in raphides in special cells called idioblasts [21] (pp. 131–144). Raphides are individually distinct, needlelike crystals that shoot out of the idioblast with high speed when the cell is disturbed (chewing), penetrating the oral mucosa and inducing immediate swelling of the lips, tongue, and oral mucosa, causing irritation/burning, vomiting, drooling, and dysphagia [2,21,72,93,94] (pp. 131–144). In severe cases, the irritative effect and lesions will extend to the pharynx, esophagus, and stomach [21] (pp. 131–144). The irritation induced by these crystals is enhanced due to the release of prostaglandins, histamine, trypsin-like proteolytic enzymes, and kinins [21,31] (pp. 131–144).

Airway obstruction and even death are possible, but life-threatening problems are rare [21,95] (pp. 131–144).

The eyes can also be affected, as contact with plant juice can induce conjunctivitis [21,96] (pp. 131–144). Dermal irritation is also possible [31].

### 2.13. Zantedeschia aethiopica

*Zantedeschia aethiopica*, or calla lily, from the *Araceae* family is native to Southern Africa and used as cut flowers or ornamental plants [2]. The calla lilies do not hold nephrotoxic properties [97]. The stem and leaves contain two phytotoxins, namely, raphides of calcium oxalate and proteolytic enzymes, which are released by the plant when damaged. Raphides are a physical irritant to the oral cavity and the esophagus, and the proteolytic enzymes induce the release of inflammatory mediators, including histamine [2,32].

Toxicosis from calla lily is reported in dogs and cats, who seem particularly susceptible [2,6,8,15], but may occur in any species [2]. Contradictions exist in the literature between true lilies and the calla lily regarding the susceptibility of cats and nephrotoxicity [98]. Therefore, there is no evidence that cats are more susceptible to calla lilies than other species.

Clinical signs manifest within 2 h post-exposure and include oral hyperemia and edema, hypersalivation, anorexia, depressed mentation, and other gastrointestinal signs, such as emesis, diarrhea, and abdominal pain; dermal exposure can cause dermatitis [2].

### 2.14. Other Ornamental Plants

Other ornamental plants involved in pet poisoning episodes in recent years in Europe are *Aucuba japonica*, *Cyclamen* spp., *Dieffenbachia* spp., *Dracaena marginata*, *Euphorbia pulcherrima*, *Ficus benjamina*, and *Rhododendron* spp. [8].

Other potentially poisonous plants for companion animals are *Adenium obesum*, *Aglaonema commutatum*, *Clivia minata*, *Codiaeum variegatum*, *Convallaria majalis*, *Crocus vernus*, *Epipremnum aureum*, *Fatsia japonica*, *Ficus* spp., *Hippeastrum x hortorum*, *Hyacinthus orientalis*, *Kalanchoe* spp., *Lonicera* spp., *Monstera deliciosa*, *Muscari armeniacum*, *Nerium oleander*, *Philodendron* spp., *Sansevieria trifasciata*, *Schefflera arboricola*, *Senecio rowleyanus*, *Syngonium podophyllum*, and *Taxus baccata* [99,100].

## 3. Conclusions

Plants are frequently involved in animal poisoning in Europe [2,8]. In recent years, emerging plant intoxications that had previously never or rarely been reported were reported in Europe; these intoxications have been associated with many aspects, including new indoor plants; environmental factors, including climate change; animal behavioral alterations; and curiosity [8]. The plants and phytotoxins presented in the current review are increasingly involved in poisoning cases around Europe, warranting an increased education and awareness of clinical veterinarians and animal owners about the origin of these emerging toxicoses, as well as the taxonomy of the identified plants and the phytotoxins they contain, to prevent toxicosis in domestic animals. Most of the emerging plant poisonings in Europe affect companion animals, especially dogs. Future research should focus on the characterization of the toxicity of these emerging phytotoxins in the newly affected species and the elaboration of effective therapeutic protocols in affected animals. The lack of a centralized reporting/poison control system in Europe leads to underreporting and nonreporting of poisoning cases in which plant toxins, emerging or not, are involved. As a consequence, information about animal poisoning is scarce to nonexistent in the vast majority of European countries. Establishing a centralized reporting system for animal poisoning cases at the European level is desirable to measure the incidence of toxicosis from emerging plants across the continent.

## Figures and Tables

**Table 1:** Toxic plants responsible for emerging plant poisonings in Europe.

Scientific Name	Common Names	Main Toxin Types	Animal Species Affected	Toxic Doses	References
*Allium cepa*; *Allium cepa* var. *aggregatum*	Onion, bulb onion, or common onion; shallot	Disulfides and thiosulfates	Dog and cat	5 g/kg of onions in cats or 15 to 30 g/kg in dogs	[17]
*Allium sativa*	Garlic	Disulfides and thiosulfates	Dog and cat	5 g/kg of garlic	[18]
*Anthurium* spp.	Flamingo plant, tailflower, or laceleaf	Insoluble calcium oxalates	Dog and cat	Irritant when chewed, irrespective of the amount	[8]
*Arum italicum*	Italian lords-and-ladies or Italian arum	Saponin (Arin), coniine-like alkaloid, insoluble calcium oxalates, and cyanogenic glycosides	Dog	Irritant when chewed, irrespective of the amount	[2,19]
*Cycas revoluta*	Sago palm or king sago	Azoxyglycosides, β-methylamino-l-alanine (BMAA), and an unidentified high-molecular-weight compound	Dog	As few as two seeds ingested by dogs can cause signs	[8,18,20]
*Lantana camara*	Red sage, yellow sage, wild sage, or shrub verbena	Pentacyclic triterpenoids	Dog	Toxicity varies considerably among cultivars, red- or yellow-flowered types are more toxic; consumption of 1% or more of the animal’s body weight is toxic	[21,22]
*Lilium* spp.	Lily	Steroidal glycoalkaloids and steroidal saponins	Cat and dog	Consumption of 1 or 2 leaves or 1 whole flower can cause toxicosis in cats	[21,23]
*Melia azedarach*	Chinaberry tree, white cedar, pride of India, or Indian lilac	Tetranortriterpenes-meliatoxins A1, A2, B1, and B2	Dog	The toxic dose is not clearly established in dogs; 5 to 6 drupes can kill a small dog	[9,24,25]
*Nandina domestica*	Nandina, sacred bamboo, or heavenly bamboo	Cyanogenic glycosides and protoberberine alkaloids	Dog and cat	The lethal dose of hydrogen cyanide is 2 to 2.5 mg/kg	[26,27]
*Persea americana*	Avocado	Acetogenins (Persin)	Dog	The toxic dose of persin is not established in dogs; foreign body obstruction in case of ingestion of a large seed	[18,21]
*Prunus laurocerasus*	Cherry laurel	Cyanogenic glycosides	Goat and dog	450 g of wilting cherry tree leaves can kill a goat; the lethal dose of hydrogen cyanide is 2 to 2.5 mg/kg	[28,29]
*Sorghum* spp.	Sorghum	Cyanogenic glycosides	Cattle	Hydrogen cyanide levels between 50 and 75 mg/100 g are considered mildly toxic and above 100 mg/100 g as extremely toxic	[30]
*Spathiphyllum* spp.	Peace lily	Insoluble calcium oxalates	Dog	Irritant when chewed, irrespective of the amount	[21,31]
*Zantedeschia aethiopica*	Calla lily	Insoluble calcium oxalates	Dog and cat, may occur in any species	Irritant when chewed, irrespective of the amount	[2,32]

## Data Availability

Not applicable.
